# Severe murine typhus complicated by multiple organ dysfunctions: A case report 

**DOI:** 10.22088/cjim.15.1.23

**Published:** 2024

**Authors:** Ermira Muco, Arta Karruli, Anjeza Dajlani, Arjana Zerja, Artan Bego

**Affiliations:** 1 Department of Infectious Diseases, Hospital University Center “Mother Teresa”, Tirana, Albania; 2 Department of Precision Medicine, University of Campania “L. Vanvitelli”, Naples, Italy; 3 Unit of Infectious Disease, Hospital of Elbasan, Albania; 4 Institute of Public Health, Tirana, Albania

**Keywords:** Rickettsial diseases, Murine typhus, Multi-organ failure

## Abstract

**Background:**

Rickettsioses are infectious diseases which are caused by intracellular bacteria which belong to the family Rickettsiaceae. This zoonosis endemically prefers tropical and subtropical regions of which the Mediterranean is included. Murine typhus is a type of rickettsial disease that commonly presents with undulating fever, headache rash, chills, malaise, and myalgias. It can lead to complications such as multi-organ failure and has a lethality rate of <5% in such cases.

**Case Presentation:**

A 70-year-old male was hospitalized at the Unit of Infectious Diseases, Mother Teresa Hospital, Tirana, Albania in a comatose condition. He had a seven-day history of fever up to 39-40°C, headache, fatigue, anorexia, vomiting, cough, and myalgia. He was a farmer and had contact with animals. Upon admission, he had scleral hemorrhages, hepatosplenomegaly, jaundice, maculopapular rash over the trunk, abdomen, and palms of his hands as well as severe acidosis, depressed bicarbonate levels, alteration in liver, kidney, and pancreas function tests. He was urgently transferred to the Intensive care unit of the Infectious Diseases Department. He was hemodynamically unstable and was put immediately on vasoactive agents and mechanical ventilation. ELISA *Rickettsia typhi* IgM resulted positive. Supportive treatment along with antibiotics Levofloxacin and Ceftriaxone was initiated. However, the patient died on the 4^th^ day of hospitalization and the 11^th^ of the disease onset.

**Conclusion:**

Murine typhus should be included in the investigation of possible causes when dealing with patients presenting with fever and maculopapular rash complicated by multi-organ failure and coming from a typhus-endemic area, especially in the summer season.

Rickettsioses are zoonoses caused by intracellular bacteria of the family Rickettsiaceae. Humans may get infected from rickettsia either by bites or contact with fluids (e.g feces) from arthropods that are infected. Rickettsial diseases are divided into 3 different groups: Typhus, spotted fever, and other rickettsial diseases. Murine typhus is a disease caused by *Rickettsia typhi* part of the typhus group (1). Flea-borne typhus which is caused by *R. typhi* and *R. felis* was an emerging infection in the USA especially in California, from 1910 to 1945 (2). Murine typhus can occur in a broad range of climacteric conditions (such as hot and humid/semiarid /cold and montane), but the highest prevalence is found in warm regions, particularly in harbor cities and seaside cities, due to the high population of rodents (3) making this zoonosis an endemic one, found mostly in seaboard regions with tropical or subtropical climacteric conditions world-wide, counting in the Mediterranean area as well (4). It is not easy to calculate this disease incidence due to its clinical symptoms which are in most cases non-specific, and therefore, most cases may go undiagnosed (5). However, the seroprevalence of endemic typhus was found to range from 1 to 20% according to several studies (6, 7). Most cases were diagnosed during summer and early autumn (8). A study by Chaliotis et al. showed that the overall cumulative incidence of this infection was 2.36 cases per 100 adult patients hospitalized with febrile disease and 0.43 per 100 adult patients hospitalized in central Greece in a 5-year timeline study (3).

Upon entering the host, rickettsiae target the endothelial cells, causing vasculitis which may cause generalized or localized clinical and laboratory findings due to the possibility to affect any organ (9). Infections caused by *Rickettsia spp* have a wide range of clinical manifestations from mild to severe forms. Murine typhus is a systemic disease characterized by rash, chills, undulating fever, headache, malaise, and myalgias. Severe forms may manifest with multi-organ failure complicated by myocarditis, nephritis, meningoencephalitis, and pneumonia (10). Although neurological involvement such as meningitis or encephalitis is considered a rare complication, a few cases have been reported (11). Diagnosing murine typhus is actually a challenge due to non-specific clinical features and the delay in serology confirmation may contribute to the delay of treatment initiation (3). The severity of the disease was found to be correlated with a delay in diagnosis and treatment, older age, organ dysfunction such as liver, renal, pulmonary, and central nervous system (4), and the lethality was <5% (12, 13). 

## Case Presentation

A 70-year-old male was hospitalized at the Unit of Infectious Diseases, Mother Teresa Hospital, Tirana, Albania in a comatose condition. He had a seven-day history of fever up to 39-40°C, headache, fatigue, anorexia, vomiting, cough, and myalgia. The patient came from Librazhd and was a farmer. His son referred that his father had been in contact with rodents (mice) and also had animals at home (cats and dogs). He did not drink alcohol and his medical history was otherwise not significant. On arrival, his temperature was 40.1°C, low blood pressure of 85/55mm Hg, pulse rate of 119/min, the respiration rate of 23/min, and oxygen saturation of 87% on room air. General examination of the patient showed scleral hemorrhages, hepatomegaly, splenomegaly, jaundice, and maculopapular rash over the trunk, abdomen, and palms of his hands. He was urgently transferred to the intensive care unit of the Infectious Diseases Department. Initial laboratory investigations showed severe acidosis, depressed bicarbonate levels, high levels of creatinine, blood urea nitrogen, alkaline phosphatase, lactate dehydrogenase, creatine kinase, bilirubin, liver enzymes, low levels of albumin, red blood cells, platelet count and normal values of white blood cells (table 1). 

**Figure 1:**
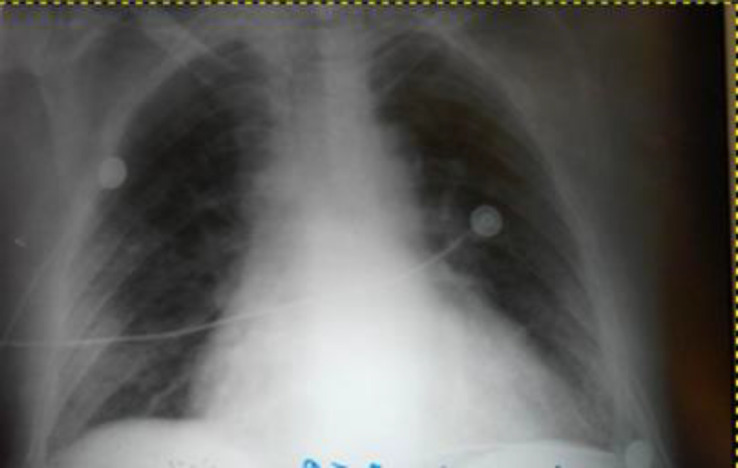
Chest radiography showed cardiomegaly, occlusion of the left phrenic-costal sinus and bilateral interstitial abnormalities

Urinalysis data showed albuminuria (1.98 gr) and microscopic hematuria. All three blood cultures taken were negative. C-reactive protein was 21.1 (normal range <0.5 mg/dL). Serological tests of *Leptospira,* *Brucella spp*., *Leishmania spp,* and viral infections such as HIV, hepatitis B and C, CMV, and EBV were negative. Protein electrophoresis and immunological examination such as ANA, C3, C4, and FR were normal. In the view of clinical history and laboratory parameters, it was suspected to be rickettsiosis from *R. typhi*, because Weil Felix test, Proteus OX19 Ag resulted- positive (1/640), ELISA *Rickettsia typhi* IgM resulted positive and ELISA *Rickettsia typhi* IgG resulted positive. Chest radiography showed cardiomegaly, occlusion of the left phrenicocostal sinus, and bilateral interstitial abnormalities (figure 1). Abdominal ultrasound showed liver 180 mm, spleen 130 mm, and a gall bladder wall thickening. The patient was treated with antibiotic therapy (Ceftriaxone and Levofloxacin, methylprednisolone, albumin, plasma, platelets, dopamine, and supportive therapy with electrolyte, vitamin, and intravenous fluids. He could not undergo treatment with doxycycline, the first-line treatment for this disease due to poor clinical conditions and the availability of only oral formulation in our institution, therefore we initiated intravenous antibiotic treatment with Levofloxacine and Ceftriaxone as they are effective against *Rickettsia spp*. 

The patient was put on mechanical ventilation immediately upon admission in addition to vasoactive agents. Despite the great professional work of Infectious disease and Intensive care specialists, his hemodynamic status worsened, the patient could not survive and died on day 4 of hospitalization and the 11th day of the disease.

**Table 1:** Hematochemical parameters progression

**Laboratory data**	**Reference range**	**Day 0**	**Day 1**	**Day 2**
**Aspartate aminotransferase**	0-35U/L	103	182	127
**Alanine aminotransferase**	0-45U/L	109	110	102
**Total bilirubin**	<1.2mg/dL	6.3	7	5.7
**Alkaline Phosphatase**	32-117U/L	319	267	292
**Amylase**	28-100U/L	121	345	1025
**Lipase**	21-67U/L	103	106	1555
**Blood albumin**	3.5-5.2g/dl	2.2	2.5	2.5
**Total proteins**	6-8.3g/dL	4.8	4.8	3.5
**Creatinine**	0.1-1.3mg/dl	5.6	5.5	5.8
**Blood urea nitrogen**	<43mg/dl	174	202	334
**Creatine kinase**	0-171U/L	335	663	1086
**Glycemia**	74-106mg/dL	127	235	192
**Platelet count**	150-390x10^3^/mm^3^	23	25	23
**White blood cells**	4000-10,000/mm^3^	9700	8200	7700
**Red blood cells**	4.7-6.1 x10^6^/mm^3^	3.62	3.74	3.78
**Hemoglobin**	13.8-17.2 g/dl	10.1	10.2	11

## Discussion

The most common Rickettsia groups in Albania are: Q fever, murine typhus, and the Mediterranean spotted fever. Our patient lived in a small city located in the southeast of Albania called Librazhd, with a latitude of 41 11' N, and a longitude of 20 19' E. In fact, as per the Köppen climate classification, Librazhd has Mediterranean climate characteristics, wet and humid in the summer. In fact, our case developed the infection in July. He was a male, which is in line with the prevalent gender evidenced by studies probably due to the highest exposure to nature due to working agricultural activities, therefore, having a high risk for contact with different rodents (14). 

Chaliotis et al. showed that even though cases occurred during all seasons, the highest peak was in the summer season due to weather favorable conditions for the rodent population to thrive and also human environmental exposure is at its highest (3). Not only rats and cats, as evidenced by most studies but also dogs are found to be reservoirs of this disease (15). The patient relatives referred that he had been in contact with rodents (mice) and also had pets (a dog and a cat) at home. We presented a case of severe murine typhus with multi-organ failure. Wachs et al. described a case of murine typhus with similar clinical features such as liver with elevated bilirubin and transaminases and kidney failure, low platelet count, and alteration in coagulation tests (16). 

The clinical signs and symptoms of our patient included fever (up to 40°C), fatigue, headache, anorexia, vomiting, cough, myalgia, scleral hemorrhages, and maculopapular rash. He manifested with hepatosplenomegaly, renal and pulmonary involvement. Laboratory exams showed severe acidosis, depressed bicarbonate levels, high levels of creatinine, blood urea nitrogen, bilirubin, alkaline phosphatase, lactate dehydrogenase, creatine kinase, liver enzymes, and low levels of albumin, platelet count, red blood cells and white blood cells. 

The maculopapular rash is a very common feature in rickettsial diseases which is also a useful clinical tool for diagnosis. However, in murine typhus, the presence of rash is variable affecting 20%-80% of patients. Murine typhus rash generally occurs 1 week after fever onset and it lasts 1-4 days. It is non-pruritic, macular, or maculopapular, usually initiating on the trunk and then gradually progressing to the periphery usually not affecting the palms and soles (4). A review study by Van der Vaar et al. (17) showed that pulmonary involvement along with symptoms such as cough were frequent features of murine typhus. Complications were found in 26.1% of patients affected by murine typhus with the most common ones being pneumonia, acute renal failure, and altered mental status. 5.9% of them were transferred to the Intensive care unit and 0.8% had multi-organ dysfunction/ septic shock (8). Acute renal failure is caused by a lack of perfusion therefore it is a pre-renal failure. Cases with acute renal failure have been described (18). Rhabdomyolysis is a rarely reported complication (19). Acute hepatitis was found in 67% of cases and hyperbilirubinemia was found in 38% (20). Factors associated with severe disease are older age, male sex, comorbidities such as liver or pulmonary diseases, and the use of alcohol or deficiency in glucose-6-phosphate dehydrogenase (G-6-PD) (21). The diagnosis of our case was based on clinical, epidemiological, and laboratory data as well as confirmation by the government health laboratory. The laboratory diagnosis of the case was conducted by serological tests of Weil-Felix test non-specific heterophile agglutination reaction of Proteus OX19 Antigen and ELISA a high sensitivity and specificity test for all rickettsial groups. It is of great importance to start oral antibiotics such as doxycycline from the first days since it is shown to be an effective treatment for this intracellular pathogen. Moreover, mortality was associated with the delay in diagnosis and treatment, with a mortality rate of 4% without proper antibiotic treatment and 1% with appropriate antibiotic treatment (4).

This disease is endemic in our country, but most cases present as mild forms with nonspecific symptoms therefore, may go undiagnosed and since complications/mortality is associated with delay in treatment patients may present at the hospital with febrile multi-organ dysfunction which is a clinical manifestation of a broad spectrum of diseases. We described this case to rise concern that this infection can develop into a severe form with non-specific signs such as multi-organ failure and when dealing with patients who come from endemic regions for murine typhus physicians need to investigate also for this infection in the differential diagnosis of the causes of multi-organ failure. 
